# A Nation-Wide Study of Prevalence and Risk Factors for Fecal Impaction in Nursing Homes

**DOI:** 10.1371/journal.pone.0105281

**Published:** 2014-08-22

**Authors:** Enrique Rey, Marta Barcelo, Maria Jose Jiménez Cebrián, Angel Alvarez-Sanchez, Manuel Diaz-Rubio, Alberto Lopez Rocha

**Affiliations:** 1 Division of Digestive Diseases, Hospital Clinico San Carlos, Universidad Complutense, Instituto de Investigacion Sanitaria San Carlos (IdISSC), Madrid, Spain; 2 Centro Valdeluz, Madrid, Spain; 3 Spanish Society of Nursing Homes Physicians (Sociedad Española de Medicos de Residencias – SEMER), Madrid, Spain; Cardiff University, United Kingdom

## Abstract

**Background:**

There are no existing studies that provide data regarding the epidemiology of, and risk factors for, fecal impaction, either in the general population or in any sub-group of people.

**Objective:**

Estimate the prevalence of and factors associated with fecal impaction on a representative sample of the institutionalized elderly population.

**Design:**

Two-phase study. Phase 1: pilot study validating the methodology in which all residents of a single nursing home participated. Phase 2: national multi-center cross-sectional study.

**Setting:**

34 randomly selected nursing homes.

**Measurements:**

The presence of fecal impaction and associated factors were evaluated using three different tools: data collected from medical records; a self-completion questionnaire filled out by the subjects or a proxy; and a rectal examination.

**Subjects:**

Older subjects living in nursing homes.

**Results:**

The prevalence of chronic constipation was 70.7% (95%CI: 67.3–74.1%), of which 95.9% of patients were properly diagnosed and 43.1% were properly controlled. The prevalence of FI according to patient history was 47.3% (43.6–51.0%) and 6.6% (4.7–8.5%) according to rectal examination. Controlled constipation (OR: 9.8 [5.2–18.4]) and uncontrolled constipation (OR: 37.21 [19.7–70.1]), the number of medications (OR: 1.2 [1.1–1.3]), reduced functional capacity (OR: 0.98 [0.97–0.99]) and the occasional use of NSAIDs were independent risk factors for fecal impaction.

**Conclusions:**

Constipation affects more than 70% of people living in nursing homes. Although it is properly diagnosed in more than 95% of cases, the disease is only controlled in less than 50%. Constipation, especially when not controlled, is the most significant risk factor leading to fecal impaction, which is prevalent in almost 50% of this population.

## Background

The increase in life expectancy has led to a rise in the proportion of older people living in developed countries; in Europe, 17.1% of the population were over 65 years-old in 2008 and this is expected to rise to 23.5% in 2030 [1]. Older people require more assistance and care, which may be delivered at home, however sometimes a nursing home is necessary. In the USA, there were 17,000 nursing homes and 1.5 million residents in 2004 [2]. The need of long term care facilities has grown; according to a report from the Organisation for Economic Co-operation and Development (OECD), between 2000 and 2009 the number of beds in nursing homes grew in most countries, reaching an average of 44 per 1,000 inhabitants over 65 and this is expected to continue to grow [3].

Constipation is a condition characterized by infrequent or difficult defecation, and is one of the most common medical problems in institutionalized people. It is estimated to affect up to 80% of this population [4]–[6], given the multiple concurrent risk factors for constipation such as immobility, multiple medications, co-morbidity, and cognitive decline. The high prevalence of constipation in institutionalized elderly patients results in not only a reduced quality of life [7], [8] and high economic burden [9]–[11], but it is also associated with the potentially serious complication of fecal impaction [12]–[14].

Although a definition of fecal impaction is elusive [15], it usually refers to the accumulation of hard feces in the rectum and colon that the subject cannot evacuate alone. There are no existing studies that provide data regarding the prevalence, incidence or risk factors for fecal impaction, either in the general population or in any subgroup of people. However, indirect data suggest that it is highly prevalent among institutionalized elderly patients, with 20% of those with fecal incontinence being diagnosed with the condition within a year [16], a prevalence of 25% in those with urinary dysfunction [17], 55% in those with diarrhea [18], and a description of stercoral ulcers (caused by fecal impaction) in autopsies of 1.3–5.7% of this population [19].

The following are considered risk factors for fecal impaction: certain medications including stimulant laxatives, immobility, neurological diseases such as Parkinson's or dementia, low fiber intake, chronic kidney failure, diabetes, or the existence of a malign neoplasm in any location [20], but there are no specific studies to support this.

The objective of this study is to estimate the prevalence of fecal impaction in a representative sample of the elderly institutionalized population, and to evaluate the risk factors associated with experiencing fecal impaction.

## Materials and Methods

### Design and population

The study was performed in two phases. The first phase consisted of a pilot study to validate the methodology, in which all residents of a single nursing home were invited to participate; the results of the validation have been published previously [21]. Once the first phase was complete and the methods were validated, the second phase was carried out: this was a national multi-center cross-sectional study. For the second phase, 34 nursing homes were selected at random from the SEMER (Sociedad Española de Médicos de Residencias [Spanish Society of Nursing Home Physicians]) members list, geographically proportional to the only estimation of the Spanish nursing home population [22]. For that purpose, physicians associated with the SEMER at the time of the study were classified into three geographical areas and chosen randomly by an officer (not related to SEMER) according to a prior specified geographical quota. When the physician either worked in the same nursing home of a previously selected physician, or was not reached (contact information not updated or impossibility to contact him/her directly after three calls on different days), an alternative physician was chosen in the same way.

Each nursing home physician was invited to include 25 residents in the study. Given that it was not possible to obtain lists of residents to form a purely random sample (due to national data protection laws), the residents of each nursing home were selected semi-randomly in accordance with pre-defined quotas according to the initial of the resident's surname and their year of birth (even/odd).

### Ethical aspects

The study was approved by the Clinical Research Ethics Committee at Hospital Clinico San Carlos, and all of the study's participants or their legal representatives signed an informed consent document prior to their participation.

### Protocol

The data gathering protocol included:

#### Clinical records abstraction

Clinical history (medical and nursing) data were gathered for each resident using a closed form that collected data on specific co-morbidities (detailed in Table 1), habitual or occasional use of several medications (detailed in Table 1), and the diagnosis of constipation. Specifically, data were gathered on diagnoses of fecal impaction in the last year, as well as its frequency and the treatment options used to resolve it.

**Table 1:** Comparison between the pilot sample and the general sample.

	Pilot Sample (N = 199)	General Sample (N = 488)
**Age**		
≤80 years old	32 (16.1%)	130 (26.6%)
81–90 years old	113 (56.8%)	246 (50.4%)
>90 years old	54 (27.1%)	112 (23%)*
**Gender [female N (%)]**	144 (72.4%)	336 (68.9%)
**Marital status [widow; N (%)]**	127 (63.8%)	265 (56.5%)
**Educational level [primary or less; N (%)]**	139 (69.8%)	400 (81.9%)*
**Time of stay in nursing home**		
<1 year	8 (4%)	11 (2.3%)
1–10 years	76 (38.4%)	91 (18.8%)
>10 years	114 (57.6%)	382 (78.9%)*
**Cognitive and Functional Status**		
Functional disability (Barthel Index; score)	64.5±28.8 (0–100)	61.3±31.3 (0–100)
Cognitive impairment (Folstein Test; score)	23.0±8.4 (0–35)	22.1±8.6 (0–35)
**Nutritional Status**		
Body Mass Index (kg/m^2^)	24.1±4.5 (14.9–43.3)	25.7±4.5 (14.7–41.5)*
Modified Ward Index (score)	5.5±5.7 (0–22)	3.6±4.9 (0–24)*
Fibre intake (g/day)	10.6±3.6 (3.9–21.9)	9.1±3.6 (2.5–24.4)*
Liquids intake (l/day)	1.5±0.3 (0.8–2.2)	1.6±0.5 (0.4–3.5)
**Physical activity**		
Immobility [bed-chair; N (%)]	56 (28.1%)	135 (28.7%)
Moderate or more [>60 min/day; N (%)]	31 (15.6%)	52 (12%)
**Metodologic**		
Needed proxy for completing [N (%)]	160 (80.4%)	384 (81.4%)
Understood every question [N (%)]	142 (71.4%)	364 (77.6%)
**Co-Morbidity**		
Diabetes [N (%)]	57 (28.6%)	129 (26.4%)
Thyroid disease [N (%)]	22 (11.1%)	55 (11.3%)
High blood pressure [N (%)]	132 (66.3%)	270 (55.3%)*
Cardiovascular diseases [N (%)]	100 (50.3%)	174 (35.6%)*
Respiratory diseases [N (%)]	50 (25.1%)	125 (25.6%)
Parkinson's disease [N (%)]	10 (5.0%)	36 (7.3%)
Stroke [N (%)]	37 (18.6%)	111 (23.1%)
Other Neurological diseases [N (%)]	78 (39.2%)	208 (42.6%)
Osteoarthritis [N (%)]	117 (58.8%)	302 (61.9%)
Renal/urinary disease [N (%)]	35 (17.6%)	66 (13.5%)
Depression [N (%)]	59 (29.6%)	200 (41.0%)*
Other psychiatric diseases [N (%)]	2 (1%)	44 (9.0%)*
Abdominal/abdominal wall Surgery [N (%)]	39 (19.6%)	110 (22.5%)
**Number of Co-morbidities per subject**	2.84±1.32 (0–7)	2.97±1.55 (0–8)
**Drugs**		
Antihypertensive drugs [N (%)]	147 (73.9%)	256 (52.4%)*
SSRIs [N (%)]	69 (34.7%)	176 (36.1%)
Tryciclic antidepressant drugs [N (%)]	7 (3.5%)	12 (2.5%)
Benzodiazepines [N (%)]	63 (31.7%)	130 (26.3%)
Hypolipemic drugs [N (%)]	53 (26.6%)	117 (24.0%)
PPIs [N (%)]	131 (65.8%)	277 (56.8%)
Calcium channel blockers [N (%)]	27 (13.6%)	53 (10.9%)
Nitrates [N (%)]	11 (5.5%)	48 (9.8%)
NSAIDs [N (%)]	26 (13.1%)	53 (10.9%)
ASA [N (%)]	68 (34.2%)	155 (31.8%)
Opiates [N (%)]	37 (18.6%)	25 (5.1%)*
Diuretics [N (%)]	84 (42.2%)	174 (35.6%)
Hypnotic drugs [N (%)]	52 (26.6%)	102 (20.9%)
**Number of drugs per subject**	4.56±2.29 (0–12)	4.02±2.20 (0–12)
**Constipation**		
Medical Diagnosis [N (%)]	119 (59.8%)	319 (65.4%)
Rome III Criteria [N (%)]	74 (37.2%)	183 (40.0%)
**Regular laxatives**		
Regular use of laxatives [N (%)]	114 (57.3%)	321 (65.8%)*
Regular use of enemas [N (%)]	4 (2.0%)	13 (2.7%)
**Fecal Impaction**		
**Recurrent fecal impaction**	62 (31.1%)	136 (27.8%)
**Fecal impaction according to rectal examination**	9 (4.5%)	35 (7.2%)

*p<0.05.

#### Subject reported information

Each participating resident completed, with the help of a proxy if necessary, a questionnaire that included the items on abdominal and defecatory symptoms from the Rome III [23] questionnaire and on nutritional information, using the Spanish version of the Ward questionnaire [24]. For the analysis, the Ward score was modified, eliminating the item regarding the need for help with cooking, since there is a central dining room at each nursing home and none of the subjects cook for him or herself.

In addition, information was also collected on some lifestyle habits (liquid intake, fiber intake, physical exercise) with an ad-hoc questionnaire. All subjects were asked to record the average number of glasses or cups of liquid (including water, soda, soup, etc.) they drank daily during the morning, lunch, evening, dinner, and night. The daily intake of liquids was calculated as the sum of these amounts, estimating 0.2 liters per glass/cup. Fiber intake was calculated with a simplified food frequency questionnaire, including six questions referring to the usual weekly intake of fruits (1 question), vegetables (2 questions), cereals (1 question), legumes (1 question), and nuts (1 question). Answers were categorized as less than one ration weekly, 1 to 3 rations weekly, 3 to 6 rations weekly, one ration daily, and 2 or more rations daily. For the analyses, these categories were summarized as 0.5, 2, 4.5, 7 and 14 rations weekly, respectively. Total fiber intake was calculated assuming 2.5 g per ration of fruits, 3.5 g for vegetables, 1 g for cereals, 3 g for legumes and 1.5 g for nuts, and expressed as grams daily. To estimate physical activity, subjects were asked to describe their usual physical activity under one of the following items: “practice sports regularly”, “walk long distances”, “walk short distances (around my house)”, “do not walk anywhere, or just a little bit”, and classified for the analysis in three categories.

#### Objective measurements

All patients were evaluated in terms of functional capacity using the Barthel test [25] and cognitive capacity using the Lobo version of the Folstein mini-mental state exam [26], [27]. Folstein's test is a widely used method to detect cognitive impairment. It is a questionnaire that evaluates temporal and spatial orientation, attention span, concentration and memory, capacity for abstraction (calculation), language ability, and visuospatial perception and ability to follow basic instructions. Validated Spanish Lobo's version provides a score ranging 0–35; 25 points or more indicates normal cognitive ability, 20 to 24 points a mild cognitive impairment, 15 to 19 a moderate impairment and 14 or less a severe cognitive impairment. Barthel's Index is a generic measure assessing the level of functional capacity independence) of the subjects for some basic activities of daily living, Each activity is evaluated by the physician with different prespecified scores according to the capacity of the examined subject to carry out these activities. The overall score ranges between 0 (completely dependent) to 100 points (completely independent). A score of 100 means complete functional capacity (independence), 90 to 99 good functional capacity (low dependence), 60 to 90 moderate functional capacity (moderate dependence), 20 to 60 low functional capacity (severe dependence), and 0 to 20 implies complete dependence.

In addition, the physician conducted a rectal examination (within two weeks of completion of the self-reported questionnaires), on all residents except those who did not consent to it. The physician was required to categorize the characteristics of the feces into one of the following categories: absence of feces, soft feces, non-impacted hard feces, and impacted feces. All physicians were provided with an information leaflet on the technique for carrying out a rectal examination and on the categorization to be used.

### Definitions

A resident was considered to experience chronic constipation when he or she was diagnosed by the physician with constipation or, having not been diagnosed with chronic constipation in their clinical history, he or she complained of sufficient symptoms to meet the Rome III criteria for chronic constipation on the intestinal symptoms questionnaire. Constipation was further categorized as “uncontrolled constipation” when, on the Rome III intestinal symptoms questionnaire, the resident complained of sufficient symptoms to meet the Rome III criteria for chronic constipation, and as “controlled constipation” when the resident did not report sufficient symptoms to meet Rome III criteria despite having being diagnosed with constipation.

Fecal impaction was defined as the existence of a hard mass of feces in the rectum which the subject was unable expel. To estimate annual prevalence of fecal impaction we defined it as the medical diagnosis of fecal impaction as registered in the medical or nurse record of the subject in the last year, with recurring impaction being defined as a record of at least two episodes in the last year. Fecal impaction was defined on the rectal examination when the physician described the feces as hard and impacted. We applied these two definitions in the same population. Since fecal impaction is an intermittent event, the first definition was intended to estimate the annual prevalence of fecal impaction while the diagnosis through rectal examination was intended to estimate how many subjects are impacted at the same time.

Fecal incontinence was defined as the involuntary loss of liquid or solid stools occurring at least once monthly according to responses to a self-report questionnaire.

### Analysis

Prevalences are reported as relative frequency accompanied by their 95% confidence interval. Quantitative variables are expressed as mean (standard deviation). Univariate analysis was used to evaluate possible risk factors associated with fecal impaction, and the factors associated with fecal impaction in that univariate analysis were subsequently included in a multivariate logistic regression (forward stepwise). Missing data were treated as missing.

### Sample size

The sample size was set at 863 subjects, which allowed for the estimation of a prevalence of 10% with 2% precision.

## Results

### Response rate

80% (N = 199) of the residents of a nursing home participated in the pilot study, and 21 of the 34 (61.7%; N = 488) invited nursing homes participated in the second phase, with an average of 25 subjects per site. There were no differences between the characteristics of the nursing homes that participated and those that did not. The participating nursing homes had capacity for 3302 residents (average: 157, range: 40–236) and 5 homes had a quality certificate issued because of the quality of their services. The 13 sites not participating had a total of 1736 residents (average: 134; range: 16–233) and one had a services quality certificate.

There were no relevant differences between the samples from the pilot study and from the general study, either in terms of the participants' socio-demographic, cognitive or functional traits, or in their medical characteristics (co-morbidities, use of medications), or in the prevalence of constipation, impaction, or the degree of control over constipation (Table 1). The proportion of subjects needing a proxy to complete a questionnaire was the same in both samples (80.4% vs 81.4%). We therefore believe that the general sample is representative of the nursing home population in Spain, and the samples were pooled for the final analysis, yielding a sample size of 687 subjects for analysis.

### Prevalence of chronic constipation

The prevalence of chronic constipation was 70.7% (95%CI: 67.3–74.1%). In total, 466 subjects were diagnosed with constipation (67.8%; 95%CI: 64.3–71.3%) and 20 (2.9%; 95%CI: 1.7–4.2%) had symptoms of constipation sufficient to meet the Rome III criteria, although they had not been diagnosed with constipation in their medical history.

Of the residents with chronic constipation that filled out the symptoms questionnaire properly (N = 458), 201 (43.1%) were adequately controlled and 257 (52.9%) were not adequately controlled.

### Prevalence of laxative consumption

63.3% (95%CI: 59.7–66.9%) of the subjects took laxatives on a regular basis. 53 (7.7%; 95%CI: 5.7–9.7%) subjects took bulk-producing laxatives, 313 (45.6%; 95%CI: 41.8–49.3%) took osmotic laxatives and 69 (10.0%; 95%CI: 7.8–12.3%) took other laxatives or combinations of laxatives. (Table 2) shows the relationship between the laxative guidelines followed and the control of constipation.

**Table 2:** Association between the laxative treatment used and the constipation control level.

	N*	Controlled Constipation (N = 201)	Uncontrolled Constipation (N = 257)
No laxatives	23	6 (3%)	17 (6.6%)
Laxatives only occasionally	26	9 (4.5%)	17 (6.6%)
Bulk-forming laxatives	51	31 (15.4%)	20 (7.8%)
Osmotic laxatives**	293	128 (63.7%)	165 (64.2%)
Other laxatives or combinations	65	27 (13.4%)	38 (14.8%)

*28 subjects without symptomatic data to classify constipation control;

**260 (88.7%) were using lactulose and 33 (11.3%) were using PEG.

### Prevalence of fecal impaction

**Figure 1:**
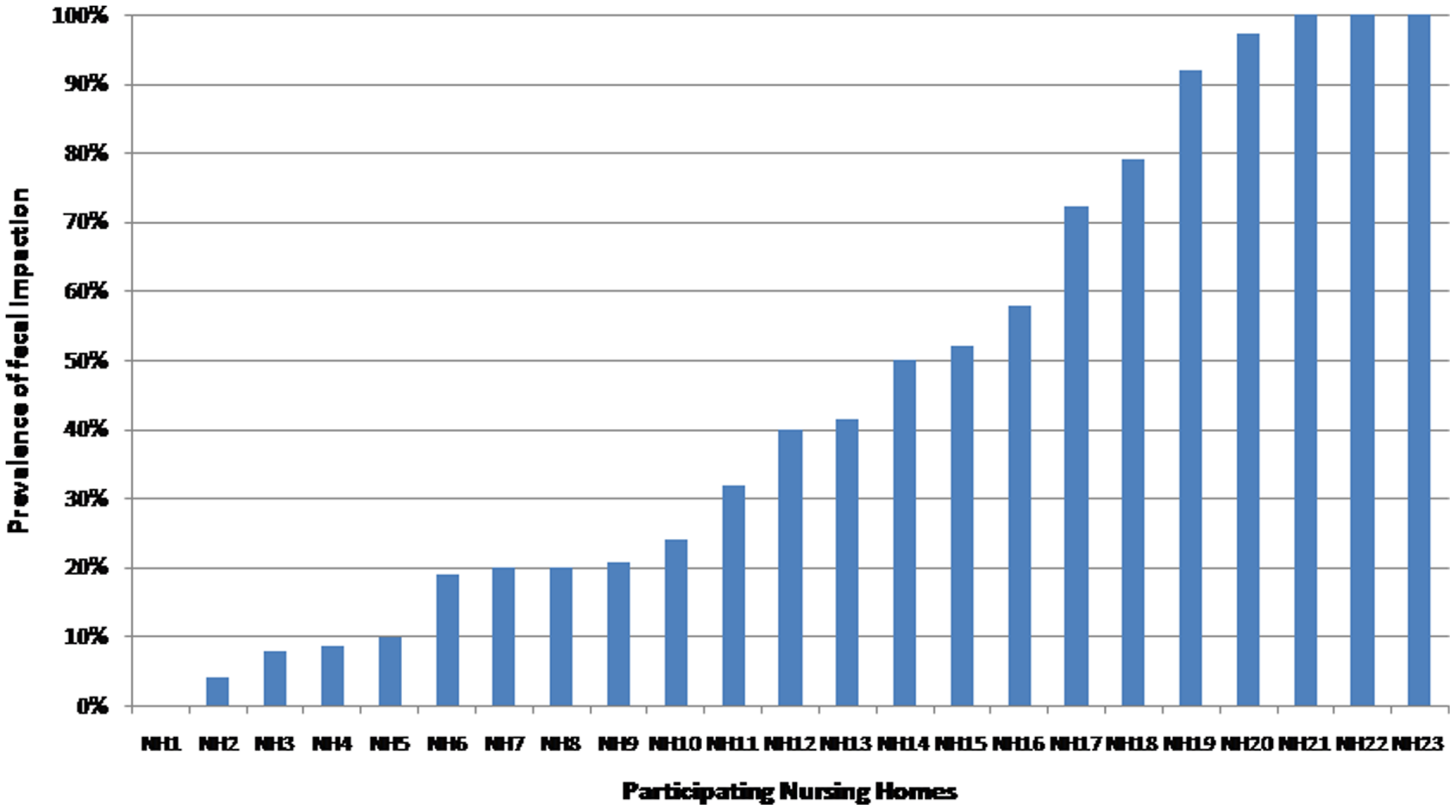
Annual prevalence of fecal impaction across the participating nursing hours.

According to their medical history, 325 subjects had experienced at least one episode of fecal impaction during the last year, which represents an annual prevalence of fecal impaction of 47.3% (95%CI: 43.6–51.0%). Variability of the prevalence of fecal impaction among participating nursing homes is shown in Figure 1. Of those with fecal impaction, 127 (18.5%) had experienced a single episode, 173 (25.2%) had experienced more than one episode but less than one per month, and 25 (3.6%) had experienced at least one episode per month. The prevalence of recurring fecal impaction was therefore 28.8% (95%CI: 25.4–32.2%).

Of the 665 (96.8%) residents that underwent rectal examination, 169 had non-impacted hard feces and 44 had impacted hard feces in the rectum, representing a prevalence of 6.6% (95%CI: 4.7–8.5%).

### Methods to solve fecal impaction

**Figure 2:**
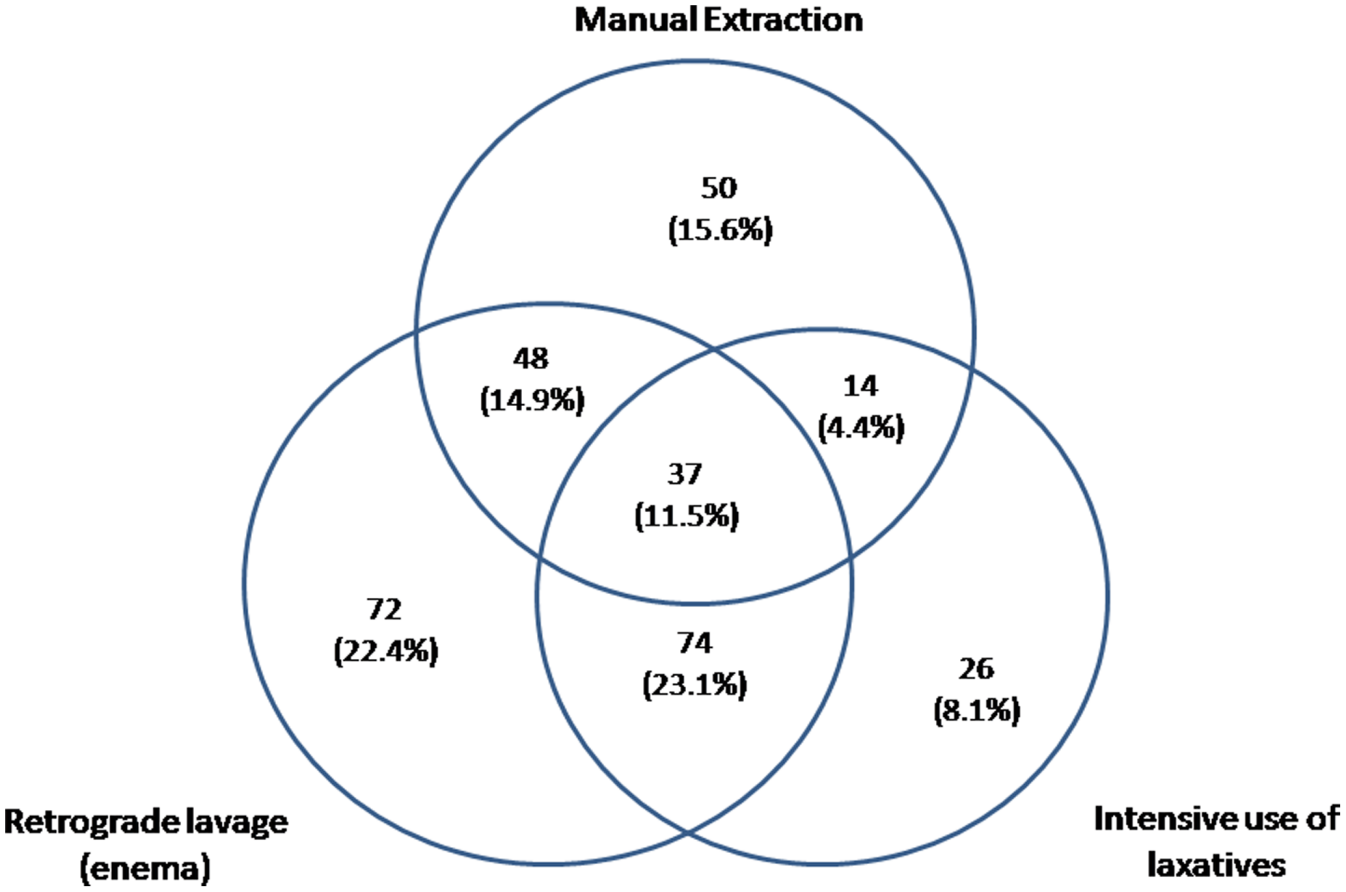
Methods used to solve fecal impaction in the population studied.

Manual extraction was used in 151 persons, retrograde lavage was used in 236, and intensive use of laxatives in 155. Figure 2 shows how these therapeutic resources were used in the sample.

### Chronic constipation and fecal impaction

The prevalence of fecal impaction, both according to the patient's medical history and present on rectal examination, was clearly related to constipation and the lack of adequate control of this (p<0.001; chi-squared test) (Table 3).

**Table 3:** Fecal Impaction and control of chronic constipation.

	Faecal impaction (medical records)	OR*	Faecal impaction (rectal examination)	OR*
No Constipation	15 (7.5%)	1	1 (0.5%)	1
Controlled constipation	96 (47.8%)	11.1 (6.1–20.1)	5 (2.5%)	4.9 (0.5–42.7)
Uncontrolled constipation	196 (76.3%)	39.2 (21.5–71.6)	35 (13.6%)	30.7 (4.2–226.4)

*adjusted by age and gender.

### Fecal impaction and fecal incontinence

Prevalence of fecal incontinence was 16.4% (59 of 359) among those without history of fecal impaction and 28.2% (88 of 312) among those with history of fecal impaction (p<0.001; chi-2).

### Factors associated with fecal impaction

In the univariate analysis, multiple factors (Table 4) were associated with the risk of having experienced fecal impaction in accordance with the diagnosis gathered from the medical history.

**Table 4:** Factors associated to Fecal Impaction.

	N	No Impaction	Impaction	OR not adjusted	OR adjusted*
**Age (years)**		83.6 (8.5)	85.1 (7.8)	**1.02 (1.00–1.04)**	
**Gender**					
Male	207	125 (60.4%)	82 (39.6%)	1	
Female	480	237 (49.4%)	243 (50.6%)	**1.56 (1.12–2.18)**	
**Body Mass Index (kg/m2)**		25.8 (4.4)	24.7 (4.5)	**0.94 (0.91–0.98)**	
**Time of staying in nursing home (months)**		38.4 (47.5)	43.9 (41.3)	1.00 (1.00–1.01)	
**Functional Status (Barthel Index; score)**		68.7 (29.0)	55.1 (30.7)	**0.99 (0.98–0.99)**	**0.98 (0.97–0.99)**
**Cognitive Status (Folstein Test; score)**		22.0 (8.9)	22.8 (8.2)	1.01 (0.99–1.03)	
**Modified Ward Index (score)**		3.3 (4.6)	5.2 (5.7)	**1.07 (1.04–1.11)**	
**Fiber intake (g/day)**		9.8 (3.9)	9.3 (3.3)	0.97 (0.93–1.01)	
**Liquids intake (l/day)**		1.6 (0.5)	1.5 (0.4)	0.90 (0.64–1.27)	
**Physical activity**					
Sport or long walks	71	53 (74.6%)	18 (25.4%)	1	
Light or moderate	407	230 (56.5%)	177 (43.5%)	**2.27 (1.28–4.00)**	
Minimum	191	76 (39.8%)	115 (60.2%)	**4.46 (2.43–8.18)**	
**Number of co-morbidities**		2.7+/−1.4	3.2+−1.5	**1.23 (1.11–1.36)**	
**Diabetes**					
No	501	256 (51.1%)	245 (48.9%)		
Yes	186	106 (57.0%)	80 (43.0%)	0.79 (0.56–1.11)	
**Thyroid**					
No	610	328 (53.8%)	282 (46.2%)		
Yes	77	34 (44.2%)	43 (55.8%)	1.47 (0.91–2.37)	
**High blood pressure**					
No	285	173 (60.7%)	112 (39.3%)		
Yes	402	189 (47.0%)	213 (53.0%)	**1.74 (1.28–2.37)**	
**Cardiovascular diseases**					
No	413	231 (55.9%)	182 (44.1%)		
Yes	274	131 (47.8%)	143 (52.2%)	**1.39 (1.02–1.88)**	
**Respiratory diseases**					
No	512	286 (55.9%)	226 (44.1%)		
Yes	175	76 (43.4%)	99 (56.6%)	**1.65 (1.17–2.33)**	
**Parkinson's disease**					
No	641	340 (53.0%)	301 (47.0%)		
Yes	46	22 (47.8%)	24 (52.2%)	1.23 (0.68–2.24)	
**Stroke**					
No	539	288 (53.4%)	251 (46.6%)		
Yes	148	74 (50.0%)	74 (50.0%)	1.15 (0.80–1.65)	
**Other Neurological diseases**					
No	400	212 (53.0%)	188 (47.0%)		
Yes	287	150 (52.3%)	137 (47.7%)	1.03 (0.76–1.40)	
**Osteoarthritis**					
No	268	168 (62.7%)	100 (37.3%)		
Yes	419	194 (46.3%)	225 (53.7%)	**1.95 (1.42–2.67)**	
**Renal/Urinary diseases**					
No	586	314 (53.6%)	272 (46.4%)		
Yes	101	48 (47.5%)	53 (52.5%)	1.27 (0.84–1.95)	
**Constipation**					
No	201	186 (92.5%)	15 (7.5%)	1	
Controlled	201	105 (52.2%)	96 (47.8%)	**11.33 (6.26–20.54)**	**9.8 (5.2–18.4)**
Uncontrolled	257	61 (23.7%)	196 (76.3%)	**39.84 (21.88–72.56)**	**37.21 (19.7–70.4)**
**Depression**					
No	428	242 (56.5%)	186 (43.5%)		
Yes	259	120 (46.3%)	139 (53.7%)	**1.51 (1.11–2.06)**	
**Psychiatric-Other**					
No	641	330 (51.5%)	311 (48.5%)		
Yes	46	32 (69.6%)	14 (30.4%)	**0.46 (0.24–0.89)**	
**Abdominal Surgery**					
No	548	292 (53.3%)	256 (46.7%)		
Yes	139	70 (50.4%)	69 (49.6%)	1.12 (0.77–1.63)	
**Number of drugs**		3.6+/−2.1	4.8+/−2.2	**1.29 (1.20–1.39)**	**1.2 (1.1–1.3)**
**Antihypertensive**					
No	281	168 (59.8%)	113 (40.2%)	1	
Regular	403	193 (47.9%)	210 (52.1%)	**1.62 (1.19–2.20)**	
Occasional	3	1 (33.3%)	2 (66.7%)	2.97 (0.27–33.18)	
**SSRIs**					
No	441	249 (56.5%)	192 (43.5%)	1	
Regular	245	113 (46.1%)	132 (53.9%)	**1.51 (1.11–2.07)**	
Occasional	1	0 (0.0%)	1 (100.0%)		
**Tricyclic Antidepressant**					
No	668	352 (52.7%)	316 (47.3%)	1	
Regular	19	10 (52.6%)	9 (47.4%)	1.00 (0.40–2.50)	
**Benzodiazepine**					
No	488	267 (54.7%)	221 (45.3%)	1	
Regular	193	94 (48.7%)	99 (51.3%)	1.27 (0.91–1.78)	
Occasional	6	1 (16.7%)	5 (83.3%)	6.04 (0.70–52.09)	
**Hypolipemics**					
No	515	276 (53.6%)	239 (46.4%)	1	
Regular	170	85 (50.0%)	85 (50.0%)	1.15 (0.82–1.63)	
Occasional	2	1 (50.0%)	1 (50.0%)	1.15 (0.07–18.56)	
**PPIs**					
No	258	159 (61.6%)	99 (38.4%)	1	
Regular	408	198 (48.5%)	210 (51.5%)	**1.70 (1.24–2.34)**	
Occasional	21	5 (23.8%)	16 (76.2%)	**5.14 (1.83–14.47)**	
**Antacids**					
No	655	350 (53.4%)	305 (46.6%)	1	
Regular	25	9 (36.0%)	16 (64.0%)	2.04 (0.89–4.68)	
Occasional	7	3 (42.9%)	4 (57.1%)	1.53 (0.34–6.89)	
**Calcium channel blockers**					
No	607	322 (53.0%)	285 (47.0%)	1	
Regular	80	40 (50.0%)	40 (50.0%)	1.13 (0.71–1.80)	
**Nitrates**					
No	627	346 (55.2%)	281 (44.8%)	1	
Regular	59	15 (25.4%)	44 (74.6%)	**3.61 (1.97–6.63)**	
Occasional	1	1 (100.0%)	0 (0.0%)		
**NSAID**					
No	517	297 (57.4%)	220 (42.6%)	1	
Regular	79	32 (40.5%)	47 (59.5%)	**1.98 (1.22–3.21)**	**1.7 (0.9–3.2)**
Occasional	91	33 (36.3%)	58 (63.7%)	**2.37 (1.50–3.76)**	**2.3 (1.2–4.5)**
**ASA**					
No	463	269 (58.1%)	194 (41.9%)	1	
Regular	223	93 (41.7%)	130 (58.3%)	**1.94 (1.40–2.68)**	
Occasional	1	0 (0.0%)	1 (100.0%)		
**Opiates**					
No	620	342 (55.2%)	278 (44.8%)	1	
Regular	62	18 (29.0%)	44 (71.0%)	**3.01 (1.70–5.32)**	
Occasional	5	2 (40.0%)	3 (60.0%)	1.85 (0.31–11.12)	
**Antidiarrheals**					
No	672	350 (52.1%)	322 (47.9%)	1	
Regular	5	4 (80.0%)	1 (20.0%)	0.27 (0.03–2.44)	
Occasional	10	8 (80.0%)	2 (20.0%)	0.27 (0.06–1.29)	
**Anticholinergics**					
No	628	332 (52.9%)	296 (47.1%)	1	
Habitual	59	30 (50.8%)	29 (49.2%)	1.08 (0.64–1.85)	
**Diuretics**					
No	406	236 (58.1%)	170 (41.9%)	1	
Regular	258	116 (45.0%)	142 (55.0%)	**1.70 (1.24–2.33)**	
Occasional	23	10 (43.5%)	13 (56.5%)	1.80 (0.77–4.21)	
**Phenothiazines**					
No	650	334 (51.4%)	316 (48.6%)	1	
Regular	37	28 (75.7%)	9 (24.3%)	**0.34 (0.16–0.73)**	
**Hypnotics**					
No	507	272 (53.6%)	235 (46.4%)	1	
Regular	155	80 (51.6%)	75 (48.4%)	1.09 (0.76–1.56)	
Occasional	25	10 (40.0%)	15 (60.0%)	1.74 (0.77–3.94)	

Nagelkerke R^2^ = 0.49.

When the variables associated with fecal impaction in the univariate analysis were included in a final multivariate logistic regression model (forward stepwise), the factors independently associated with fecal impaction were controlled constipation (OR: 9.8 [5.2–18.4]) and uncontrolled constipation (OR: 37.21 [19.7–70.1]), the number of medications (OR: 1.2 [1.1–1.3]), reduced functional capacity (OR: 0.98 [0.97–0.99]) and the occasional use of NSAIDs (OR: 2.3 [1.2-4-5]) (Table 4).

### Factors associated with fecal impaction in the rectal examination

In the univariate analysis, multiple factors (Table 5) were associated with the risk of having impacted feces in the rectum during the rectal examination.

**Table 5:** Factors associated to Fecal Impaction in rectal examination.

	N	No Impaction	Impaction	OR not adjusted
**Age (years)**	687	84.2 (8.2)	86,3 (7.3)	1.04 (0.99–1.08)
**Gender**				
Male	207	195 (94.2%)	12 (5.8%)	1
Female	480	448 (93.3%)	32 (6.7%)	1.16 (0.59–2.30)
**Body Mass Index (kg/m^2^)**	643	25.2 (4.5)	26.0 (4.2)	1.04 (0.97–1.11)
**Time of staying in nursing home (months)**	682	40.3 (45.0)	50.6 (39.9)	**1.00 (1.00–1.01)**
**Functional Status** (Barthel Index; score)	642	63.3 (30.3)	46,4 (30.5)	**0.98 (0.97–0.99)**
**Cognitive Status** (Folstein Test; score)	673	22.3 (8.6)	23.6 (7.0)	1.02 (0.98–1.06)
**Modified Ward Index (score)**	687	3.9 (5.2)	7.8 (5.0)	**1.12 (1.07–1.17)**
**Fibre intake (g/day)**	665	9.6 (3.6)	8.3 (4.2)	**0.89 (0.80–0.98)**
**Liquids intake (l/day)**	664	1.6 (0.4)	1.3 (0.5)	**0.29 (0.13–0.63)**
**Physical activity**				
Sport or long walks	71	70 (98.6%)	1 (1.4%)	1
Light or moderate	407	378 (92.9%)	29 (7.1%)	5.37 (0.72–40.07)
Minimal	191	179 (93.7%)	12 (6.3%)	4.69 (0.60–36.77)
**Number of co-morbidities**	687	2.8 (1.4)	4.3 (18.8)	**1.77 (1.46–2.15)**
**Diabetes**				
No	501	476 (95.0%)	25 (5.0%)	1
Yes	186	167 (89.8%)	19 (10.2%)	**2.17 (1.16–4.04)**
**Thyroid diseases**				
No	610	575 (94.3%)	35 (5.7%)	1
Yes	77	68 (88.3%)	9 (11.7%)	**2.17 (1.00–4.72)**
**High blood pressure**				
No	285	274 (96.1%)	11 (3.9%)	1
Yes	402	369 (91.8%)	33 (8.2%)	**2.23 (1.11–4.49)**
**Cardiovascular diseases**				
No	413	399 (96.6%)	14 (3.4%)	1
Yes	274	244 (89.1%)	30 (10.9%)	**3.50 (1.82–6.74)**
**Respiratory diseases**				
No	512	496 (96.9%)	16 (3.1%)	1
Yes	175	147 (84.0%)	28 (16.0%)	**5.90 (3.11–11.21)**
**Parkinson's Disease**				
No	641	598 (93.3%)	43 (6,7%)	1
Yes	46	45 (97.8%)	1 (2.2%)	0.31 (0.04–2.30)
**Stroke**				
No	539	506 (93.9%)	33 (6.1%)	1
Yes	148	137 (92.6%)	11 (7.4%)	1.23 (0.61–2.50)
**Other Neurological diseases**				
No	400	372 (93.0%)	28 (7.0%)	1
Yes	287	271 (94.4%)	16 (5.6%)	0.78 (0.42–1.48)
**Osteoarthritis**				
No	268	259 (96.6%)	9 (3.4%)	1
Yes	419	384 (91.6%)	35 (8.4%)	**2.62 (1.24–5.55)**
**Renal/Urinary Diseases**				
No	586	551 (94.0%)	35 (6.0%)	1
Yes	101	92 (91.1%)	9 (8.9%)	1.54 (0.72–3.31)
**Constipation**				
No	201	200 (99.5%)	1 (0.5%)	1
**Controlled**	201	196 (97.5%)	5 (2.5%)	**5.10 (0.59–44.07)**
**Uncontrolled**	257	222 (86.4%)	35 (13.6%)	**31.53 (4.28–232.27)**
**Anxiety Disorder**				
No	492	469 (95.3%)	23 (4.7%)	1
Yes	195	174 (89.2%)	21 (10.8%)	**2.46 (1.33–4.56)**
**Depression**				
No	428	411 (96.0%)	17 (4.0%)	1
Yes	259	232 (89.6%)	27 (10.4%)	**2.81 (1.50–5.27)**
**Other Psychiatric diseases**				
No	641	600 (93.6%)	41 (6.4%)	1
Yes	46	43 (93.5%)	3 (6.5%)	1.02 (0.30–3.43)
**Abdominal Surgery**				
No	548	508 (92.7%)	40 (7.3%)	1
Yes	139	135 (97.1%)	4 (2.9%)	0.38 (0.13–1.07)
**Number of drugs**	687	4.0 (2.2)	6.2 (2.1)	**1.46 (1.28–1.67)**
**Antihypertensive**				
No	281	272 (96.8%)	9 (3.2%)	1
Regular	403	368 (91.3%)	35 (8.7%)	**2.87 (1.36–6.08)**
Occasional	3	3 (100.0%)	0 (0.0%)	
**SSRIs**				
No	441	422 (95.7%)	19 (4.3%)	1
Regular	245	220 (89.8%)	25 (10.2%)	**2.52 (1.36–4.68)**
Occasional	1	1 (100.0%)	0 (0.0%)	
**Tricyclic Antidepressant**				
No	668	625 (93.6%)	43 (6.4%)	1
Regular	19	18 (94.7%)	1 (5.3%)	0.81 (0.11–6.19)
**Benzodiazepines**				
No	488	460 (94.3%)	28 (5.7%)	1
Regular	193	177 (91.7%)	16 (8.3%)	1.49 (0.78–2.81)
Occasional	6	6 (100.0%)	0 (0.0%)	
**Hypolipemics**				
No	515	486 (94.4%)	29 (5.6%)	1
Regular	170	155 (91.2%)	15 (8.8%)	1.62 (0.85–3.10)
Occasional	2	2 (100.0%)	0 (0.0%)	
**PPIs**				
No	258	240 (93.0%)	18 (7.0%)	1
Regular	408	385 (94.4%)	23 (5.6%)	0.80 (0.42–1.51)
Occasional	21	18 (85.7%)	3 (14.3%)	2.22 (0.60–8.26)
**Antiacids**				
No	655	616 (94.0%)	39 (6.0%)	1
Regular	25	22 (88.0%)	3 (12.0%)	2.15 (0.62–7.51)
Occasional	7	5 (71.4%)	2 (28.6%)	**6.32 (1.19–33,61)**
**Calcium channel blockers**				
No	607	573 (94.4%)	34 (5.6%)	1
Regular	80	70 (87.5%)	10 (12.5%)	**2.41 (1.14–5.08)**
**Nitrates**				
No	627	595 (94.9%)	32 (5.1%)	1
Regular	59	47 (79.7%)	12 (20.3%)	**4.75 (2.29–9.82)**
**NSAID**				
No	517	492 (95.2%)	25 (4.8%)	1
Regular	79	68 (86.1%)	11 (13.9%)	**3.18 (1.50–6.76)**
Occasional	91	83 (91.2%)	8 (8.8%)	1.90 (0.83–4.35)
**ASA**				
No	463	449 (97.0%)	14 (3.0%)	1
Regular	223	193 (86.5%)	30 (13.5%)	**4.99 (2.59–9.61)**
**Opiates**				
No	620	581 (93.7%)	39 (6.3%)	1
Regular	62	59 (95.2%)	3 (4.8%)	0.76 (0.23–2.53)
Occasional	5	3 (60.0%)	2 (40.0%)	**9.93 (1.61–61.19)**
**Antidiarrheals**				
No	672	628 (93.5%)	44 (6.5%)	1
Regular	5	5 (100.0%)	0 (0.0%)	
Occasional	10	10 (100.0%)	0 (0.0%)	
**Anticholinergics**				
No	628	586 (93.3%)	42 (6.7%)	1
Regular	59	57 (96.6%)	2 (3.4%)	0.49 (0.12–2.08)
**Diuretics**				
No	406	396 (97.5%)	10 (2.5%)	1
Regular	258	230 (89.1%)	28 (10.9%)	**4.82 (2.30–10.11)**
Occasional	23	17 (73.9%)	6 (26.1%)	**13.98 (4.55–42.94)**
**Phenothiazines**				
No	650	607 (93.4%)	43 (6.6%)	1
Regular	37	36 (97.3%)	1 (2.7%)	0.39 (0.05–2.93)
**Hypnotics**				
No	507	474 (93.5%)	33 (6.5%)	1
Regular	155	147 (94.8%)	8 (5.2%)	0.78 (0.35–1.73)
Occasional	25	22 (88.0%)	3 (12.0%)	1.96 (0.56–6.88)

Applying a multivariate model, without including constipation in its codification, because the existence of a single case in the reference category (no constipation) made calculations impossible, and including, in its place, two categories: (1) no constipation or controlled constipation, (2) uncontrolled constipation, the factors independently associated with having impacted feces in the rectum during the rectal examination were the lack of control of constipation (OR: 11.84 [3.87–36.24]), the number of medications (OR: 1.26 [1.02–1.56]), reduced functional capacity (OR: 0.98 [0.97–0.99]), risk of malnutrition (OR: 1.14 [1.02–1.22]), the habitual use of ASA (OR: 3.12 [1.24–7.87]) and the occasional use of diuretics (OR: 18.94 [3.69–97.15]) (Table 6).

**Table 6:** Multivariate Model: factors associated with fecal impaction in rectal examination.

	P	OR adjusted*
Number of drugs	0,03	1.26 (1.02–1.56)
Functional Status (Barthel score)	0.02	0.98 (0.97–0.99)
Modified Ward index	<0.001	1.14 (1.02–1.22)
Uncontrolled constipation	<0.001	11.84 (3.87–36.24)
ASA	0.01	3.12 (1.24–7.87)
Diuretics		
No		
Regular	0.08	2.48 (0.90–6.81)
Occasional	<0.001	18.94 (3.69–97.15)

Nagelkerke R2 = 0.42.

## Discussion

This is the first study specifically designed to evaluate the prevalence of both constipation and fecal impaction in a sample representative of the nursing home population. The data available up until now came from studies that were not specifically designed for this objective, and were obtained from a single nursing home, which limits its interpretation in terms of representation, given the variation in standards of care and human and material resources among institutions.

Institutionalized elderly patients represent a very specific population, given that they can be expected to suffer constipation more frequently than the non-institutionalized elderly population due to the high prevalence of known risk factors for it [4], [6], [28]–[30], but they are under constant nursing and medical supervision. Our study provides information on the prevalence of the conditions studied, as well as on the outcome of care, and the known or supposed therapeutic and preventive measures taken against these.

Our study confirms that constipation in the nursing home population is very highly prevalent, affecting more than 70% of said population. Although there are no other studies on prevalence with which we can compare our results, this figure is similar to the one obtained in studies providing indirect data on the prevalence of constipation, such as the use of laxatives on at least an occasional basis by 93% of the residents of nursing homes [31], the daily use of laxatives by 50–74% of institutionalized persons [4]–[6], [32], or the fact that more than 50% of institutionalized patients complain of straining or difficulty passing feces in more than 25% of bowel movements [33].

Moreover, in spite of the fact that constipation is well-known and is diagnosed correctly (95.9% of patients with constipation were correctly diagnosed in the study sample), treatment efforts to control it are generally insufficient, as shown by the fact that more than 50% of patients diagnosed with constipation continue to meet the Rome III criteria for constipation, in spite of the treatment prescribed by their physicians, and in spite of being surrounded by constant care. It may suggest that laxatives are less effective in this population; an alternative explanation is that the effectiveness of laxatives is not checked after prescription. Future studies should be focused on this relevant matter, since control of constipation is the main objective of treatment.

Our study provides, for the first time, figures on the prevalence of fecal impaction in the institutionalized population, revealing the enormous magnitude of the problem and its high recurrence rate. Around 50% of residents experienced fecal impaction at least once a year, 30% experienced recurring bouts, and 6.6% were impacted at any given time when a rectal examination was performed. This occurred despite them living in institutions that guarantee daily healthcare and that provide medical supervision, in many cases with procedures submitted for external evaluation and certification. Our figures for recurring fecal impaction align greatly with those obtained in a 1975 study, in which it was revealed that 39% of patients with fecal impaction had a prior history of impaction [34]. The fact that almost 40 years later the prevalence of recurring fecal impaction has not dropped to any significant extent clearly demonstrates that the measures currently being taken to prevent its occurrence are insufficient.

Of all the factors associated with fecal impaction, our study confirms that constipation is the most significant individual factor associated with the occurrence of fecal impaction. Moreover, the results reinforce the idea that the main risk factor, specifically, is the inability to control constipation, in spite of treatment. Other factors, such as a lack of activity, the risk of malnutrition, and medications taken, are additional factors that contribute to fecal impaction in a population with a high incidence of constipation.

Of all of the identified risk factors for fecal impaction, there is one that has not previously been described in the literature: the occasional use of NSAIDs. Although this is not one of the factors most strongly associated with fecal impaction, it is relevant due to the high prevalence of NSAID use in the institutionalized elderly population. Although we know that the use of NSAIDs is a risk factor for constipation in the general population [35], our study shows that it is also a risk factor for experiencing fecal impaction that is independent of the risk for constipation.

The fact that our study does not show an association with certain factors that have been related to fecal impaction in other studies, such as neurological co-morbidities [13], or low liquid intake [20], not even in the univariate analysis, is probably related to the nursing home healthcare staff's knowledge of these risk factors for constipation, and their thus paying special attention to them in these patients.

A notable limitation of our study is the semi-random selection of the patients at each nursing home, imposed by the data protection laws, which made it impossible for us to access the patient registry for each nursing home. Although some bias in the selection of the subjects may exist in some centers, it would have occurred in both directions towards the most well and the most unwell. Overall, we believe that the consequences of this limitation are minimal, since we were able to compare the semi-randomness at a single nursing home with 80% of the residents included with random selection of nursing homes. The nursing homes included in the study are representative of all of the nursing homes in Spain, in accordance with the nursing home physician's census from the SEMER. Moreover, the response rate was lower than expected, which allowed us to reach the pre-established sample size: nevertheless, the sample size reached allows for at least 4% accuracy for the prevalence of 47% observed for impaction.

The objectivity of the self-reported measures by this population may be considered another possible limitation to our study. Although the instruments used have been previously validated for this purpose, the study was controlled by categorizing the patients according to the degree of cognitive decline, and by including only the medically defined data as an outcome for the analysis. Although 100% self-reporting would be desirable, it is impossible in this population without the aid of a proxy. In fact, more than 80% needed a proxy to complete the questionnaire mainly for two reasons: functional limitation (visual or motor impairment), and cognitive limitations for self-completing the questionnaire. More than 70% of the participants were able to fully understand the questions included in the questionnaire; some required a proxy due to reading or writing difficulties. The remainder of subjects required a proxy for completing the questionnaire due to cognitive impairment, but in these subjects the usual caregiver is likely to provide more reliable answers than the subject. The pilot study was designed in part to address this limitation by evaluating if the project was affordable.

Another limitation that should be acknowledged is that estimates of food, fluid and fiber intake are approximate, but a more accurate measure, such as a 3-day diary, was unaffordable; in the pilot study we tested that approach and it was not possible to obtain complete and reliable information.

The final conclusions of our study from a practical and clinical point of view are that fecal impaction is a problem of significant magnitude and that constipation is the most relevant associated factor. In an environment of constant medical care such as a nursing home, particular attention should be paid to ensuring that treatment for constipation is effective, regardless of the patient's capacities, habits, medical characteristics, or co-treatments.

## References

[1] Konstantinos Giannakouris (2010) Regional population projections EUROPOP2008: Most EU regions face older population profile in 2030. Eurostat. Statistic in focus. http://epp.eurostat.ec.europa.eu/portal/page/portal/product_details/publication?p_product_code=KS-SF-10-001.

[2] Jones AL, Dwyer LL, Bercovitz AR, Stahan G (2009) The National Nursing Home Survey: 2004 overview. National Center for Health Statistics Vital Health Stat 13 167: http://www.cdc.gov/nchs/data/nnhsd/nursinghomefacilities2006.pdf 19655659

[3] OECD (2011) “Long-term care beds in institutions and hospitals”, in Health at a Glance 2011: OECD Indicators, OECD Publishing. http://dx.doi.org/10.1787/health_glance-2011-72-en.

[4] Primrose WR, Capewell AE, Simpson GK, Smith RG (1987) Prescribing patterns observed in registered nursing homes and long-stay geriatric wards. Age Ageing January; 16 1: 25–8. 3105271 10.1093/ageing/16.1.25

[5] Kinnunen O (1991) Study of constipation in a geriatric hospital, day hospital, old people's home and at home. Aging (Milano) June; 3 2: 161–70. 1911905 10.1007/BF03323997

[6] Harari D, Gurwitz JH, Avorn J, Choodnovskiy I, Minaker KL (1994) Constipation: assessment and management in an institutionalized elderly population. J Am GeriatrSoc September; 42 9: 947–52. 10.1111/j.1532-5415.1994.tb06585.x 8064102

[7] O'Keefe EA, Talley NJ, Tangalos EG, Zinsmeister AR (1992) A bowel symptom questionnaire for the elderly. J Gerontol July; 47 4: M116–M121. 1624694 10.1093/geronj/47.4.m116

[8] Glia A, Lindberg G (1997) Quality of life in patients with different types of functional constipation. Scand J Gastroenterol November;32 11: 1083–9. 9399387 10.3109/00365529709002985

[9] Singh G, Lingala V, Wang H, Vadhavkar S, Kahler KH, et al (2007) Use of health care resources and cost of care for adults with constipation. Clin Gastroenterol Hepatol September; 5 9: 1053–8. 17625982 10.1016/j.cgh.2007.04.019

[10] Rao SS (2007) Constipation: evaluation and treatment of colonic and anorectal motility disorders. GastroenterolClin North Am September; 36 3: 687–711, x. 10.1016/j.gtc.2007.07.013 17950444

[11] Dennison C, Prasad M, Lloyd A, Bhattacharyya SK, Dhawan R, et al (2005) The health-related quality of life and economic burden of constipation. Pharmacoeconomics 23 5: 461–76. 15896098 10.2165/00019053-200523050-00006

[12] Kimberly BS (2007) Constipation in the elderly: implication in skilled nursing facilities. Director 15 3: 20–3. 19343856

[13] Gallagher P, O'Mahony D (2009) Constipation in old age. Best Pract Res ClinGastroenterol 23 6: 875–87. 10.1016/j.bpg.2009.09.001 19942165

[14] De Lillo AR, Rose S (2000) Functional bowel disorders in the geriatric patient: constipation, fecal impaction, and fecal incontinence. Am J Gastroenterol April; 95 4: 901–5. 10763934 10.1111/j.1572-0241.2000.01926.x

[15] Creason N, Sparks D (2000) Fecal impaction: a review. NursDiagn January; 11 1: 15–23. 10.1111/j.1744-618x.2000.tb00381.x 10847055

[16] Chassagne P, Landrin I, Neveu C, Czernichow P, Bouaniche M, et al (1999) Fecal incontinence in the institutionalized elderly: incidence, risk factors, and prognosis. Am J Med February; 106 2: 185–90. 10230748 10.1016/s0002-9343(98)00407-0

[17] Starer P, Likourezos A, Dumapit G (2000) The association of fecal impaction and urinary retention in elderly nursing home patients. Arch GerontolGeriatr January; 30 1: 47–54. 10.1016/s0167-4943(99)00051-5 15374048

[18] Kinnunen O, Jauhonen P, Salokannel J, Kivela SL (1989) Diarrhea and fecal impaction in elderly long-stay patients. Z Gerontol November; 22 6: 321–3. 2623935

[19] Lal S, Brown GN (1967) Some unusual complications of fecal impaction. Am J Proctol June; 18 3: 226–31. 6046373

[20] Wrenn K (1989) Fecal impaction. N Engl J Med September 7; 321 10: 658–62. 2671728 10.1056/NEJM198909073211007

[21] Barcelo M, Jimenez-Cebrian MJ, Diaz-Rubio M, Rocha AL, Rey E (2013) Validation of a questionnaire for assessing fecal impaction in the elderly: impact of cognitive impairment, and using a proxy. BMC Geriatr 13: 24. 23496919 10.1186/1471-2318-13-24 PMC3599666

[22] Equipo Portal Mayores (2010) Estadisticas sobre residencias: distribucion de centros y plazas residenciales por provincia. Datos de octubre. Informes Portal Mayores Núm 104 Pág: 19 p.ISSN: 1885–6780. Available: http://envejecimiento.csic.es/documentacion/biblioteca/registro.htm?id=57572.

[23] Morgan DR, Squella FE, Pena E, Mearin F, Rey E, et al (2010) Multinational validation of the Spanish Rome III Adult diagnostic questionnaire: comparable sensitivity and specificity to English instrument (abstract). Gastroenterology 138 Suppl 1: S386.

[24] Morillas J, Garcia-Talavera N, Martin-Pozuelo G, Reina AB, Zafrilla P (2006) [Detection of hyponutrition risk in non-institutionalised elderly]. NutrHosp November; 21 6: 650–6. 17147061

[25] Mahoney FI, Barthel DW (1965) Functional evaluation: the Barthel index. Md State Med J February; 14: 61–5. 14258950

[26] Lobo A, Ezquerra J, Gomez BF, Sala JM, Seva DA (1979) [Cognocitive mini-test (a simple practical test to detect intellectual changes in medical patients)]. Actas Luso EspNeurolPsiquiatrCienc Afines May; 7 3: 189–202. 474231

[27] Folstein MF, Folstein SE, McHugh PR (1975) “Mini-mental state”. A practical method for grading the cognitive state of patients for the clinician. J Psychiatr Res November; 12 3: 189–98. 1202204 10.1016/0022-3956(75)90026-6

[28] Bouras EP, Tangalos EG (2009) Chronic constipation in the elderly. GastroenterolClin North Am September; 38 3: 463–80. 10.1016/j.gtc.2009.06.001 19699408

[29] Coyne KS, Cash B, Kopp Z, Gelhorn H, Milsom I, et al (2011) The prevalence of chronic constipation and faecal incontinence among men and women with symptoms of overactive bladder. BJU Int Jan; 107 2: 254–61. 20590548 10.1111/j.1464-410X.2010.09446.x

[30] Talley NJ (2004) Definitions, epidemiology, and impact of chronic constipation. Rev GastroenterolDisord 4 Suppl 2:S3–S10. 15184814

[31] Frank L, Schmier J, Kleinman L, Siddique R, Beck C, et al (2002) Time and economic cost of constipation care in nursing homes. J Am Med Dir Assoc July; 3 4: 215–23. 12807641 10.1097/01.JAM.0000019536.75245.86

[32] Wigzell FW (1969) The health of nonagenarians. GerontolClin (Basel) 11 3: 137–44. 10.1159/000245227 5770790

[33] Marfil C, Davies GJ, Dettmar PW (2005) Laxative use and its relationship with straining in a London elderly population: free-living versus institutionalised. J Nutr Health Aging 9 3: 185–7. 15864398

[34] Gurll N, Steer M (1975) Diagnostic and therapeutic considerations for fecal impaction. Dis Colon Rectum September; 18 6: 507–11. 1081034 10.1007/BF02587220

[35] Chang JY, Locke GR, Schleck CD, Zinsmeister AR, Talley NJ (2007) Risk factors for chronic constipation and a possible role of analgesics. Neurogastroenterol Motil November; 19 11: 905–11. 17988275 10.1111/j.1365-2982.2007.00974.x

